# Indigenous-led precision public health: a new starting point

**DOI:** 10.3389/fpubh.2024.1427246

**Published:** 2024-08-29

**Authors:** Megan Fiona Baxter, Amanda Collins-Clinch, Kevin Doxzen, Yarlalu Thomas, Shahmir Rind, Vicki O’Donnell, Gareth Baynam

**Affiliations:** ^1^Wellcome Centre for Human Genetics, Oxford University, Oxford, Oxfordshire, United Kingdom; ^2^School of Medicine, Griffith University, Gold Coast, QLD, Australia; ^3^Aboriginal Health Council of WA, Perth, WA, Australia; ^4^Strategic Analysis, Inc., Arlington, VA, United States; ^5^Faculty of Health and Medical Sciences, University of Western Australia, Perth, WA, Australia; ^6^Western Australian Register of Developmental Anomalies, King Edward Memorial Hospital, Perth, WA, Australia; ^7^Faculty of Medicine, Curtin University, Perth, WA, Australia; ^8^Rare Care, Perth Children’s Hospital, Perth, WA, Australia; ^9^School of Earth and Parliamentary Sciences, Faculty of Science and Engineering, Curtin University, Perth, WA, Australia

**Keywords:** precision public healthcare, Aboriginal peoples, public health initiatives Mappa, Lyfe Languages, Pilbara Faces

## Abstract

Precision public healthcare has been applied to bring about positive change, narrowing the gap in healthcare inequity for Aboriginal peoples. Three such examples include the Mappa, Lyfe Languages, and Pilbra Faces projects, which were all developed through engagement and codesign with Indigenous Australians and each meet a distinct critical need. The Mappa project offers patients and healthcare providers with the necessary geographical information to navigate and maximally utilize available healthcare services. Lyfe Languages is a community driven translational tool that empowers indigenous languages in healthcare. The Pilbara Faces project aims to create a database of clinical measurements enabling better disease diagnosis and monitoring. These three projects have been integrated into a multi-faceted precision public health program, the Healthy Pilbara Project Initiative, acting synergistically to improve the lives of Aboriginal peoples living in Western Australia.

## 1 Introduction

Precision public health (PPH) has been defined as “using the best available data to target more effectively and efficiently interventions of all kinds to the most and to those most in need” (1, 2). PPH builds on and unites with longstanding approaches to public health, yet it’s focus on data and targeted implementation through innovative technologies makes it notably distinct. Such technologies include spatial or geographic information systems (GIS) technologies; imaging, community participation and crowdsourcing; data and analytics; and (gen)omics (3). These projects are implemented at the individual, community and systemic level to bring about lasting change.

Western Australia is more than a quarter the size of the United States of America and 10 times the size of the United Kingdom, yet has a total population of only 2.6 million people (4). This equates to an overall population density of approximately 1 person per square km with roughly 80% of individuals living within the capital city, Perth. For the remaining 20% of the population, vast distances between people and healthcare services pose significant challenges to timely access, particularly for Aboriginal communities (5, 6). In 2021, approximately 89,000 people in Western Australian, identified as Aboriginal with many residing in rural and remote communities (4). For example, in some parts of the remote Pilbara, up to 85% or more of individuals identify as Indigenous Australians (5).

Aboriginal health inequities exacerbated by remoteness has not gone unrecognized. In 2008, a federal campaign entitled *Close the Gap* was created aiming to reduce inequities across health, education and employment between Aboriginal and Torres Strait Islander peoples and Non-Indigenous Australians (7). From a healthcare perspective the *Close the Gap* campaign had two primary health related outcomes. The first of which was to close the currently existing gap in life expectancy between Aboriginal and non-Aboriginal Australians within one generation. The second was to half the current under 5 mortality rate of indigenous children within 10 years. Through a particular focus on social determinants of health the first 10 years of the campaign resulted in a 10% improvement in age standardized mortality rates across Indigenous individuals. There was however a similar mortality improvement rate across non-Indigenous individuals. This means that despite a substantial increase in life expectancy for Indigenous Australians the gap in life expectancy still unfortunately exists. The 2020 *Closing the Gap* report documented a 8.6 year gap for men and a 7.8 year gap for women, highlighting the need for alternative approaches to addressing this perpetual health inequity (8). There is a need not only for improved access to healthcare but also a need for patient centric specific healthcare, in particular in rural and remote communities.

In order to meet these needs, each of the following initiatives were co-designed with Aboriginal Western Australians (9). Briefly, key principles from best-practice co-design for Health with Indigenous people were used including: (1) Indigenous leadership (e.g., Mappa conceptualized, designed and implemented and governed by Aboriginal Controlled Health Organisations together with Aboriginal Health Providers and Community Members); (2) Culturally grounded approach (e.g., Lyfe Languages embraces cultural narratives, e.g., Songlines to explain concepts related to genetics; Mappa has dedicated focusses on local Lore and is iteratively implementing language translations through Lyfe Languages; and the Cliniface software developed through Pilbara Faces converts facial variation into text descriptors that are in translated into Aboriginal languages through Lyfe Languages); (3) Respect (e.g., honoring timelines of communities and community members in the development of Pilbara Faces); (4) Benefit to community (e.g., capacity building through employment and scholarships in precision public health and digital health, and implementation of culturally respectful health resources in clinics with Lyfe Languages); (5) Inclusive partnerships (e.g., Indigenous youth partner with their community and Elders to perform translations and to ensure that these translations are endorsed by Elders for trusted of the community, and they partner with non-Indigenous youth for further inclusivity and harmony); and (6) Transparency and evaluation (e.g., Lyfe Languages is evaluated as part of a national government funded initiative for developing resources for people living with rare diseases and the results of evaluations are made openly accessible). These initiatives were also accelerated through workshops at the inaugural Aboriginal Health Council of Western Australia co-designed Precision Public Health Asia Conference, Fremantle, Western Australia (10, 11).

**Figure 1:**
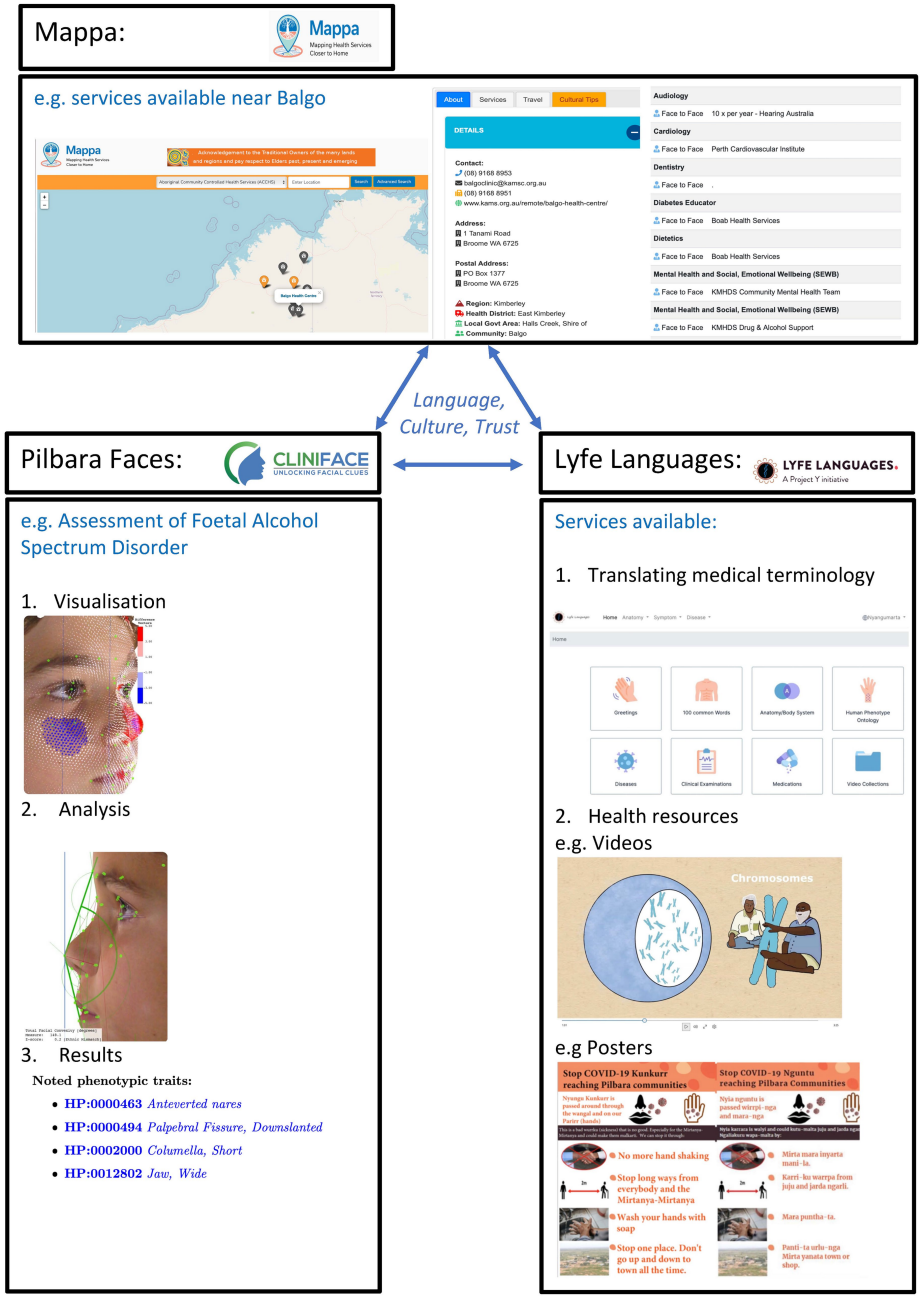
Indigenous led Precision Public Health—integrating new, old and ancient data and technology. Top panel: Mappa, mapping health services closer to home. Displays geospatial mapping of health services and other factors to support culturally safe and appropriate care. Left panel: Illustrates the Cliniface software including data analysis, visualization and reporting. In this case focused to the fetal alcohol spectrum disorder use case and incorporating Aboriginal Australian normative facial reference data. Right panel: Lyfe Languages, showing some of the functionalities, e.g., text, audio, visual representations of translations and narratives for different domains, e.g., rare diseases, COVID. All of these are united through a focus on language, culture and trust.

This paper highlights 3 interwoven initiatives responding to Indigenous community needs (see Figure 1):

1. Mappa (12).
2. Lyfe Languages (13).
3. Pilbara Faces (14).

**(1) Mappa**

Mappa is a free-to-use online mapping platform developed by the Aboriginal Health Council of Western Australia (AHCWA) and its 23 Member Services, Aboriginal Community Controlled Health Services (ACCHS).

This platform was developed to address the lack of clarity and visibility (at all levels) of health care services being delivered from a diverse range of providers across rural, remote and metropolitan regions throughout Western Australia.

The following three gaps in healthcare standards were noted during the initial design: no single health service provided information across all sectors; no cohesive repository offered curated data; and no information site (e.g., website, or register) stored data on available services. These three gaps means that collectively there is no listing of available information with regards to which primary healthcare and allied health services are available or where the closest available service is located. This gap in information significantly hinder patients’ abilities to access relevant information and understand what services are available to them and in particular in region and remote communities when these service will be next available. It is imperative that available services are being appropriately utilized and one of the primary sources of referrals is clinical recommendation. Unfortunately, in this fragmented and sparse information ecosystem, referrals and recommendations are an extremely time-consuming process for clinicians. The referral process is reliant on an availability heuristics and the manual assessment of each service individually. This means there is a clinical barrier to offer or recommend timely services and potentially an underutilization of available services.

Mappa provides comprehensive, culturally respectful and reliable information for health services, health professionals, clients, patients and their communities. The Mappa project was created to unite the patient journey, healthcare provisions, and technological innovation. Mappa is a quintessential example of PPH by enabling effective health at the right time, for the right person, and at the right place. The innovative technologies behind this project include GIS technology, connected health data sets and services, weather information, itinerary builders, and information on local cultural customs to deliver culturally respectful and more efficient care closer to home. Creators of Mappa gave particular attention to developing a friendly user interface for those who are not technologically competent and/or for whom English is not their first language.

**(2) Lyfe Languages**

There are more than 250 Indigenous languages in Australia. However none of these languages are included in large scale, freely accessible platforms such as Google Translate (15). There is also strong evidence of the importance of retaining and empowering Indigenous languages, with regards to patient wellbeing (16–18). Separately, there is also evidence that as language extinction continues to occur there will be a significant loss of unique medical knowledge (19). Lyfe Languages is an Aboriginal community-driven translational tool now being applied to rare diseases, Coronavirus Disease (COVID) and other health domains.

This project began in the Pilbara in regional Western Australia with the translation of medical terms into the Nyangumarta language. As part of this project, culturally respectful healthcare resources including posters, patient letters and animations have also been produced. Lyfe Languages is now used across a range of Indigenous Australian languages and is also active on three continents. The translations focused initially on the Human Phenotype Ontology (HPO) terms before expanding to include other relevant health terms with text, image and audio format translations produced (20). In order to enable these translations, Indigenous youth are partnered with Elders in their community to translate using digital technologies, with all translation curated by elders and translations performed culturally endorsed, and forming part of a multigenerational program. For transparency and to promote trust the names of the translator and curating Elders are visible against each translation. This process retains Indigenous language and empowers by connecting communities through new technology and ensuring the transmission of language across generations. It also continues to make healthcare more accessible and equitable, ensuing appropriate access and healthcare literacy for all Australians.

**(3) Pilbara Faces**

One third of people living with a rare disease have diagnostic facial signatures, many of which are subtle (21). Historically, identification of these facial cues was reliant on manual measurement and subjective assessment of the clinical gestalt. Increasingly, 3D facial analysis has been used to support diagnosis and monitoring (22, 23). Use of 3D facial analysis requires understanding normal versus disease-specific facial variation. Some facial variation is however population specific, which can limit the use or efficacy of 3D facial analysis across many communities. The Pilbara Faces project is iteratively improving 3D facial reference data improving reliability of Cliniface software (24).

Cliniface is an open source, free to use platform that performs local analysis and can evaluate against statistic norms for different demographics. To overcome the lack of diversity of reference data on which to develop equitable biomedical innovation, and specifically the lack of non-Caucasian 3D facial reference data, the Cliniface team and its international partners have developed, and are further progressing, a range of reference range data for multiple populations, e.g., Japanese, Chinese, Malay and Indian peoples. To support inclusivity, the Pilbara Faces initiative was focussed to commence overcoming the lack of Aboriginal 3D facial reference data. The Cliniface data is derived from a diverse range of Aboriginal communities to provide a basis for the most appropriate assessments. This project aims to ensure that Aboriginal people could better benefit from a range of applications of Cliniface. These applications include advancing screening and diagnosis (e.g., rare diseases, the initial focus domain of Cliniface; sleep disordered breathing and Fetal Alcohol Spectrum disorder, the later in response to needs identified by Aboriginal communities and the lack of pre-existing lack of tools tailored specifically for Aboriginal facial variation); treatment, and clinical trial assessment (e.g., lysosomal storage disorders); and personalized facial device design and assessment (e.g., for newborns in the nursery, COVID). Overall, this is reducing the number of invasive tests required and decreasing the need for medical travel, which is important for those living in rural and remote regions.

Each of these three initiatives are working synergistically to bring about positive change within Aboriginal communities. For example, Lyfe Languages is embedded in Mappa and also embedded in Cliniface for the generation of patient reports in Aboriginal language. Further, the Healthy Pilbara Project Initiative combined Lyfe Languages, Pilbara Faces and clinical whole genome sequencing for pre-and post-test genetic counseling unifying and improving the diagnostic process for patients.

## 2 Future directions

These initiatives provide the initial foundation to bring about much needed change, however further work is still needed to expand the impact of Precision Public Health to not only benefit the most, but also those most at need. Overall, it is critical to better embrace holistic, coordinated and integrated approaches that traverse all sectors that are necessary for improved well-being, e.g., health, education, disability, social services, community and environment. For example, Lyfe Languages is extending from its initial focus on health, to also address needs in education and social services. It is imperative to embedded language, culture, community, connection to land, sea and skies as well as social and emotional well-being in approaches to health and well-being. Lyfe Languages is therefore being integrated with a new model of cross-sector (e.g., health, education, disability and social services) care coordination at the Rare Care Centre at Perth Children’s Hospital. The Rare Care Centre uses the Aboriginal Controlled Health Organisation Model of Care at its foundation for care of all children (Aboriginal and non-Aboriginal) seen through its clinical service. The Rare Care Centre will increasingly partner with the Mappa platform to support care closer to home.

Awareness is imperative to support the expanding knowledge, design and implementation that is critical to Precision Public Healthcares continued success. The community engagement, co-design and co-implementation of Pilbara Faces started with a focus on raising awareness of, and addressing the unmet needs in rare diseases, and its uses are increasingly expanding across a range of common disease domains It is critical to prioritize the development of regulatory processes and health technology assessments (HTA) that are more aligned and responsive to patient, family and community needs, ensuring that key stakeholders remain at the centre of the design and implementation of programs. A future development of Lyfe Languages is developing a module for clinical trials and novel therapies. This will include a focus on rare disease trials and treatments to overcome known barriers to inclusion and access that in turn have regulatory and HTA implications.

From a data perspective it necessary to generate further reference data sets across a range of underrepresented populations (phenomic, e.g., 3D facial phenotype data, and other omics) to overcome, and hopefully even leapfrog, the current divide in the utility of biomedical innovation. Likewise, it is of particular benefit to expand the focus to include primary prevention, circumventing key health issues from ever arising. In order to facilitate this it is imperative to increasing the prioritization and funding for community led health and research priority setting, co-design and co-implementation.

## 3 Conclusion

A suite of Precision Public Health initiatives have been co-designed with Indigenous Australians and implemented to serve unmet needs that arose to serve unmet needs in remote areas, but that also have applications to those living in metropolitan regions. These initiatives provide a new starting point on which to build equity and scale for both rare and common disorders. These initiatives support a two-way learning approach poised to benefit Indigenous and non-Indigenous people.

## Data Availability

The original contributions presented in the study are included in the article/supplementary material, further inquiries can be directed to the corresponding author.
